# Ophthalmology

**Published:** 1876-04

**Authors:** 


### III. Ophthalmology.

1. *Tattooing the Cornea.* Dr. Howe. (*Transact. Med. Soc., County of Erie.*)

At the fifty-fifth annual meeting of the "Medical Society of the County of Erie," Dr. Howe presented an essay on the operation of tattooing the cornea. (By the way, "*cornu punction*," which the author suggested as a more descriptive name, is neither more descriptive than tattooing, nor is it a very felicitous construction, since "cornu" and "cornea" are the names of two entirely different objects.)

The operation which first has been cultivated methodically by Wecker, of Paris, is very simple; it consists in pricking a little coloring matter into the corneal substance. The principal object of this procedure is its cosmetic effect in disguising an unsightly white spot (leucoma) in the cornea; if the blemish is in the centre of the cornea, opposite the pupil, it is to be changed into a black spot; if it is in a peripheric portion of the cornea, it ought to be given the color of the iris as nearly as possible. As to the coloring matter, it must be permanent, and its use must not be attended by any subsequent irritation of the cornea. As for a black color, Indian ink is the best substance; the various tints of the iris may be imitated by the use of blue ink, or any mixture of blue and black, black and red, or all three combined. Wecker used a single broad needle, with a groove on one side, for pricking the coloring substance (previously rubbed down with water to a thick paste) into the cornea. A number of modifications have since been suggested and used, the principal object being to increase the effectiveness by multiplying the needles. But though theoretically it appears perfectly correct to increase the number of pricking points in order to save time to the surgeon and pain to the patient, yet practically this multiplication is very limited, because, while a single needle will readily puncture a substance, a number closely together act like one blunt surface. The author therefore recommended an instrument which consisted of two ordinary needles placed side by side, and having a guard so near their extremity as to prevent their passing through the cornea. With most ophthalmic surgeons this precautionary stop near the points of the needle might perhaps be unnecessary, but it seemed anyhow advisable, since such accidents as piercing the cornea had happened in the author's experiments, and also in Guy's Hospital. The operation can not be done on every eye disfigured by a leucoma; in order to be safe and successful, and not to be followed by a dangerous inflammation of the cornea or iris, these rules must be observed:

1. That we do not attempt the coloring of too large an extent either at one sitting, or altogether.

2. That the eye be free from any inflammation.

3. That the relative position of the various tissues be not so altered as to have an inflammation readily induced, *e. g.*, extensive adhesions of the iris to the corneal cicatrix.

2. *A Case of Daily Recurring Total Blindness of One Eye.* DR. L. KOENIGSTEIN. (*Klin. Monatsbl.*, *Sept.*, 1875.)

June 29, Dr. K. was consulted by a lady who gave the following history: Two evenings ago, towards six o'clock, she suddenly noticed a dimness coming over her right eye, and covering the left eye she could see absolutely nothing. This monocular blindness still continued when she retired, but on next morning the sight of the right eye was just as perfect again as ever, and continued so until six o'clock P. M., when the blindness recurred as on the preceding eve. The lady aged 31 years, in good health, has never had any serious illness. An examination failed to discover any marked difference in the external or internal appearance of the two eyes, excepting that the visual power of the right eye was somewhat weaker than that of the left. At 6 P. M., the patient felt a violent tearing pain in the supraorbital region, attended by black rings passing over the right eye from the temple toward the nose; these rings steadily grew larger until they filled the whole right side of the visual field. This entire change took about half a minute. Pressure on the supraorbital region was painful. The right eye did not perceive even the light thrown into it by the ophthalmoscope, but the right pupil responded to the light as readily as the left; and the ophthalmoscope also then failed to reveal any morbid condition which could account for the total amaurosis. Patient had a sound sleep, but on request she stayed awake that night, and about one o'clock she remarked to the doctor that she felt as if a black cloth were being removed from her right eye. And while a few minutes ago the right eye was totally blind, it then could discern light from shade; after ten minutes she could locate the light of a candle at a great distance, and after a short time she began to see the objects in the room.

The patient was ordered ten grains of quinine daily, in three powders, one to be taken half an hour before the attack, one during the same, and the third after it. Under this treatment the amaurosis set in later every day, while the sight returned regularly at one o'clock; and finally, on July 24, the amaurosis occurred for the last time at 12 P. M., to return no more. That the "blind spells" gradually grew shorter and at last disappeared after the administration of quinine does not seem to be a matter of mere coincidence, as the following schedule may show:

June 27, 28, 29, 30. Expectant treatment, amaurosis began regularly at 6 P. M.

| | | | | | | | | | | |
|---|---|---|---|---|---|---|---|---|---|---|
| July | 1, | 2, | 3, | 4, | 5, | 6, | 7, | 8, | 9. | Quinine, ten grains daily. |
| Amaurosis at | 6.30, | 7, | 7.30 | 8, | 8.30, | 9, | 9, | 9, | 9. | |

| | | | | | | | | |
|---|---|---|---|---|---|---|---|---|
| July | 10, | 11, | 12, | 13, | 14, | 15, | 16. | Quinine, 20 grs. daily. |
| Amaurosis at | 9.15 | 9.30, | 9.45, | 10, | — | — | 11. | |

July 17, 18, 19, 20, always at 11 P. M., until dose of quinine was increased to thirty grains pro die.

| | | | | |
|---|---|---|---|---|
| July | 21, | 22, | 23, | 24. |
| Amaurosis at | 11.15, | 11.30, | 11.45, | 12. |

3. *Ophthalmia Neonatorum.* S. C. AYRES. (*Cincinnati Lancet and Observer*, 1876, No. 1.)

In order to illustrate the importance of an early treatment of this disease, the author gives the statistics of one hundred cases treated in private practice, and of one hundred cases treated in the Cincinnati Hospital. We might reasonably expect, in the class of patients which we find in the hospitals, to have more trouble in the management of such a disease as purulent conjunctivitis than we would have in private practice. But the result of his statistics shows that in not a single one of the hundred hospital cases did any corneal complication arise, and all recovered with good vision, while in private practice 58 cases only were free from corneal complication and recovered with perfect vision, while in 42 the integrity of the eye was more or less impaired ; six of them were hopelessly blind in both eyes, and five in one eye, from ulceration and sloughing of the cornea. The treatment pursued in both classes of cases being practically the same (cleansing, instillations of solution of alum or silver every hour, and application of a solution of nitrate of silver gr. v to xx ad oz. j every morning), the better result obtained in the hospital can be attributed to the fact only that the inflamed eyes of the infants receive attention and treatment *immediately*, while in private practice they are too often neglected by the parents and by the family physician too. And yet there is nothing in the treatment which any intelligent physician could not carry out successfully. "If purulent conjunctivitis were treated as promptly and carefully as other infantile diseases are, we would have fewer blind in our asylums, and much less suffering and distress to the world."

4. *Castor Oil as a Menstruum for Dissolving Atropia for Application to the Eye.* DR. J. GREEN. (*Trans. Am. Ophthalmol. Soc.*, 1875.)

"Dissolve one grain of atropia in two minims of strongest alcohol, and mix with fresh castor oil, in any quantity, from half a drachm or less to an ounce, according to the strength desired. It is well to expose the stronger solutions for a short time to a gentle heat in a water bath, in order to drive off a part of the alcohol, which otherwise renders the application somewhat irritating to the surface of the eye. In this way it is easy to prepare a clear and permanent solution. I have found it to be practically unirritating to the abraded or inflamed cornea, and I believe that it is occasionally to be preferred to the aqueous solution of the sulphate. It has seemed to be most useful in recent abrasions and painful phlyctenulæ and ulcers of the cornea, with or without iritis. In case of photophobia, with excessive flow of tears, I have often applied it to the conjunctival surface of the everted upper lid—a plan which seemed to ensure a longer contact of the remedy with the cornea than is the case when aqueous solutions are dropped into the conjunctival sac."

